# Supplementary material for: Genome-Wide Identification of lncRNAs During Rice Seed Development
Source: Genes (Basel). 2020 Feb 26;11(3):243. doi: 10.3390/genes11030243 (PMC7140839; doi:10.3390/genes11030243)
Supplement: Supplementary file 1 [file genes-11-00243-s001.zip › Table S2.docx]

**Table S2 Summary of RNA-seq data for each sample.**

| **Seed**  **sample** | **Read Number** | **GC content** | **≥Q30(%)** | **Mapped reads** | **Percentage** |
| --- | --- | --- | --- | --- | --- |
| 0 DAP | 80320146 | 53.28 | 85.47 | 66244442 | 82.48% |
| 3 DAP | 65898564 | 54.18 | 87.58 | 51740405 | 78.52% |
| 7 DAP | 90564588 | 55.21 | 84.10 | 75466513 | 83.33% |

^Footnote: Read Number, Single-ended total reads number in Clean data; Base Number, Total number of bases in Clean data; GC content, GC content in Clean data; Q30, probability of base identification error is 1/1000. Percentage represents alignment efficiency of sequencing read with reference genome. DAP, Day After Pollination.^
